# Are We Neglecting Nutrition in UK Medical Training? A Quantitative Analysis of Nutrition-Related Education in Postgraduate Medical Training Curriculums

**DOI:** 10.3390/nu13030957

**Published:** 2021-03-16

**Authors:** Laura Ganis, Tatiana Christides

**Affiliations:** 1 Barts Health NHS Trust, London E1 1BB, UK; 2 Leicester Medical School, University of Leicester, Leicester LE1 7HA, UK; tc243@leicester.ac.uk

**Keywords:** medical education, postgraduate, training, curriculum, communication, nutrition, diet, obesity

## Abstract

Suboptimal nutrition is a major cause of morbidity and mortality in the United Kingdom (UK). Although patients cite physicians as trusted information sources on diet and weight loss, studies suggest that the management of nutrition-related disorders is hindered by insufficient medical education and training. Objectives of this study were to: (1) Quantify nutrition-related learning objectives (NLOs) in UK postgraduate medical training curriculums and assess variation across specialties; (2) assess inclusion of nutrition-related modules; (3) assess the extent to which NLOs are knowledge-, skill-, or behaviour-based, and in which Good Medical Practice (GMP) Domain(s) they fall. 43 current postgraduate curriculums, approved by the General Medical Council (GMC) and representing a spectrum of patient-facing training pathways in the UK, were included. NLOs were identified using four keywords: ‘nutrition’, ‘diet’, ‘obesity’, and ‘lifestyle’. Where a keyword was used in a titled section followed by a number of objectives, this was designated as a ‘module’. Where possible, NLOs were coded with the information to address objective 3. A median of 15 NLOs (mean 24) were identified per curriculum. Eleven specialties (25.6%) had five or less NLOs identified, including General Practice. Surgical curriculums had a higher number of NLOs compared with medical (median 30 and 8.5, respectively), as well as a higher inclusion rate of nutrition-related modules (100% of curriculums versus 34.4%, respectively). 52.9% of NLOs were knowledge-based, 34.9% skill-based, and 12.2% behaviour-based. The most common GMP Domain assigned to NLOs was Domain 1: Knowledge, Skills and Performance (53.0%), followed by Domain 2: Safety and Quality (20.6%), 3: Communication, Partnership and Teamwork (18.7%), and 4: Maintaining Trust (7.7%). This study demonstrates considerable variability in the number of nutrition-related learning objectives in UK postgraduate medical training. As insufficient nutrition education and training may underlie inadequate doctor-patient discussions, the results of this analysis suggest a need for further evaluation of nutrition-related competencies in postgraduate training.

## 1. Introduction

In the United Kingdom (UK), malnutrition—defined as overnutrition and undernutrition—is common [1]. A third of children leaving primary school, and 63% of adults, are now classified as overweight (Body Mass Index, BMI > 25 kg/m^2^) or obese (BMI > 30 kg/m^2^) [2,3], and approximately 5% of the population are underweight (BMI < 20 kg/m^2^) [4]. Individuals admitted to hospital are more likely to be underweight or morbidly obese (>40 kg/m^2^) compared to the general population [5], and the cost for disease-related malnutrition is over GBP 13 billion [1]. Despite this, malnutrition is often under-recognised and under-treated [1,4,5].

Professional regulatory bodies’ guidelines for UK clinicians recognise the importance of healthcare professionals in addressing nutrition and diet [6,7,8,9,10]. The Royal College of Physicians states that doctors have a responsibility to address nutrition-related issues in healthcare [8], and the General Medical Council (GMC) affirms that medical students and doctors should be able to discuss the role of nutrition in health and assess patients’ diet and weight [6,7]. The National Health Service (NHS) highlights nutrition training in their action plan for the prevention of ill health and health inequalities, “making sure staff on the frontline who are in contact with thousands of patients a year feel equipped to talk to them about nutrition and achieving a healthy weight in an informed and sensitive way” [3]. Most importantly, patients cite their physicians as trusted information sources regarding healthful food choices and weight loss [11,12]. 

Clinicians, however, are under-prepared to facilitate informed nutrition-related decision-making with patients—a finding substantiated by studies worldwide [13,14,15]. Inadequate nutrition education in medical school is commonly cited as an obstacle in the engagement of doctors with diet and weight-related interventions. In the UK, a recent study of 853 medical students and doctors found that over 70% had received less than two hours nutrition training while at medical school [15]. In addition, lack of knowledge about nutrition guidelines, unawareness of effective clinical communication tools for discussing nutrition and weight management with patients, and a lack of physician leadership/role models have been identified as barriers to physicians providing nutrition counselling [13,15,16,17].

Undergraduate nutrition-related medical education forms an essential foundation for trainee doctors, but continued learning and professional development is required throughout each stage of postgraduate training [18]. Learning objectives are set out within training programme curriculums, which outline what trainees will be taught, trained, and assessed on. The Generic Professional Capabilities (GPC) Framework [19]—which separates competencies into either knowledge-, skill- or behaviour-based outcomes [18,20]—is mapped to learning objectives. In addition, Good Medical Practice (GMP) Domains [18]—which outline the standards expected of a doctor within four distinct domains—are also embedded into curriculums. This includes Domain 1: Knowledge, Skills and Performance; Domain 2: Safety and Quality; Domain 3: Communication, Partnership and Teamwork; and, Domain 4: Maintaining Trust. Unlike the GPC Framework, learning objectives may be designated more than one GMP Domain. The creation of objectives, and their designation to the GPC and GMP Domain frameworks, is the responsibility of the Royal Colleges and affiliated bodies. Curriculums are then approved by the GMC [20]. Learning and development are assessed in training via workplace-based assessments and postgraduate examinations against these objectives [20]. 

Similar to undergraduate programmes, however, deficiencies in nutrition-related education across UK postgraduate medical training have been identified for over 20 years [21,22]. Until the early 1990s, the majority of doctors had not received nutrition-related education, either in undergraduate or postgraduate training [23]. Even as nutrition learning objectives have come to be integrated into curriculums, their significance is often undermined by the disjointed and disorganised way the objectives are included [24,25]; without recognition of nutrition as a discrete clinical specialty, or a ‘nutrition system’ equal to the cardiovascular, respiratory and other conventionally defined systems, curriculums fail to provide a cohesive picture of the role of nutrition in medical education and training [25]. This problem has only been exacerbated by the fact that nutrition remains championless amongst medical professionals, with many clinical teachers and physician role models themselves having had little or no training in nutrition [24]. 

Various initiatives, such as the Nutrition Task Force and Intercollegiate Group on Nutrition (ICGN), have been trialled to improve awareness and knowledge around nutrition in medicine, recognising the need for systematic education and training in nutrition as a prerequisite to safe and competent clinical practice [22]. The Nutrition Task Force was established in 1994 to develop a core nutrition curriculum for undergraduate medical students [26], bringing together learning outcomes in a coherent way; it also included 18 generic objectives for doctors, focusing on principles of nutritional science, public health, and clinical nutrition (see Appendix A). There is no evidence, however, to suggest that these generic objectives were ever systematically incorporated into postgraduate training. In 1996, the ICGN was formed with the specific aim of improving nutrition-related postgraduate education and training through the implementation of a fundamental nutrition course, aimed at all doctors regardless of their specialty [22,27]. In a 2012 report from the House of Commons [22], the ICGN’s ‘Intercollegiate Course in Human Nutrition’ had run a total of 22 times over 15 years. Consequently, whilst these strategies highlight the importance of nutrition-related education for doctors in training, their variable implementation and inconsistent uptake have largely left doctors with the fragmented and unsystematic approach to nutrition-related learning objectives common to both undergraduate and postgraduate curriculums [25]. 

Survey responses from doctors across the country recognise the deficit of nutrition education in postgraduate medical training [15]—but, to our knowledge, there is no published data on the current outcomes of nutrition-related education in UK postgraduate training programmes. The aim of this study was to carry out a quantitative, critical synthesis of nutrition-related education in current, GMC-approved postgraduate curriculums by identifying nutrition-related learning objectives (NLOs) and their designation within the GPC and GMP Domain frameworks. The extent to which current curriculums organise NLOs into cohesive modules, rather than sporadic inclusion of objectives within other systems, was also assessed. 

## 2. Materials and Methods

### 2.1. Materials

Between August and October 2020, one investigator (L.G.) independently reviewed the published curriculums of 43 UK postgraduate medical training programmes. Only approved curriculums, published on the GMC website as the most up-to-date, current version, were included in the analysis. One exception to this was the curriculum for the Foundation Training Programme, which was obtained from the Foundation Programme website in a similar standardised format. Six Core training pathways were included in this study: the Foundation Programme, Acute Care Common Stem (ACCS), Core Medical Training (CMT), Core Psychiatry Training, Core Surgical Training (CST), and Internal Medical Training (IMT) Stage 1. Although IMT replaced the CMT programme as of August 2019, both were included in the analysis as trainees gaining core competencies in medicine may be on either curriculum until 2021. The additional Specialty curriculums included in this analysis were chosen to represent a broad spectrum of patient-facing postgraduate medical training programmes in the UK (GMC-approved specialty curriculums not included in this study available in Appendix B).

### 2.2. Identifying Nutrition-Related Learning Objectives

Potential keywords to identify nutrition-related objectives were explored using Medical Subject Headings (MeSH) tree structures in the National Institutes of Health (NIH) database. Using this method, over 50 potential descriptors were found (a non-exhaustive list of the potential keywords identified is available Appendix C). 

Potential keywords were disregarded if they were:

- Too narrow to be included as a useful keyword, e.g., meat, salivation, edible plants, gastroenterostomy;
- Too broad to be included as a useful keyword, e.g., digestion, biometry. However, all broad terms were explored for more specific qualification using the branching MeSH tree structure, e.g., biometry→anthropometry;
- Associated with another keyword/descriptor to the extent that they reliably appear within its search or are irrelevant without the coexisting descriptor, e.g., (weight) *loss*, (nutrition)*al supplements*, *parenteral* (nutrition), (appetite) *regulation*, (feed)*ing behaviour*, *healthy* (diet).

Using this approach, three overarching keywords were chosen: ‘nutrition’, ‘diet’, and ‘obesity’. ‘Lifestyle’, which can refer to nutrition, exercise, stress, substance abuse, sleep, and social relationships [28], was also included as an intended “catch-all”, to avoid missing more broadly defined objectives. 

To identify NLOs, curriculums were searched for the four keywords—‘nutrition’, ‘diet’, ‘obesity’, and ‘lifestyle’—using the Find function (ctrl + F). Keywords that formed a stem for another word or phrase, e.g., nutrition (al assessment), diet(ician), (parenteral) nutrition, were identified using this method and included in the analysis. A pilot search demonstrated no improvement in NLO identification with the addition of other possible keyword descriptors identified in the MeSH tree structure search (Appendix C). Keywords that appeared within general text (such as the curriculum introduction) were not included in the analysis.

Identified NLOs were compiled in a Google Sheets spreadsheet and tallied. Duplicate keywords within the same objective, for example, “understand the influence of lifestyle on health and the factors that influence an individual to change their lifestyle”, were counted as one NLO. Where two different keywords were encountered within the same objective, these were tallied individually; for example, “outline the available treatments for obesity, including diet, exercise, medication and surgery”. This was done in order to acknowledge the emphasis on two related, but distinct, entities.

### 2.3. Identifying Modules

Where a keyword was used in the title of a specific section of the curriculum and followed by a number of objectives, this was designated as a ‘module’. All listed objectives within a module were tallied individually, regardless of their individual reiteration of the keyword.

### 2.4. Coding Knowledge-, Skill-, or Behaviour-Based Outcomes

Where possible, objectives were coded as knowledge-, skill-, or behaviour-based. This was done only for objectives in which the curriculum explicitly assigned this information. No attempts were made to subjectively code objectives that had not been formally categorised within the curriculum.

### 2.5. Coding Good Medical Practice (GMP) Domains

Where possible, objectives were coded with a GMP Domain (see Table 1). This was done only for objectives in which the curriculum clearly and explicitly assigned this information. Objectives that had not been formally categorised within the curriculums were not subjectively coded.

Spearman’s Rank Correlation Coefficient (*Rs*) was used to assess the relationship between keywords and GMP Domains. For each curriculum, the proportion of NLOs identified by each keyword was ranked against the proportion of NLOs designated to each Domain; for example, in the Core Medical Training curriculum, 18% of objectives were ‘obesity’ NLOs, and 16% of NLOs were assigned to Domain 3. The Correlation Coefficient and critical probability (*p*) value were calculated using a Spearman’s Rank Calculator [available online at https://www.socscistatistics.com/tests/spearman/default2.aspx; accessed 1 March 2021]. The *Rs* value was calculated using the following standard formula:

$$Rs=1-\;\left(\frac{6\Sigma d^{2}}{n^{3}-n}\right)$$

### 2.6. Communication-Based Objective Search

An additional search was carried out to identify communication-based competencies within identified NLOs; that is, those NLOs referring specifically to communication, counselling, behaviour modification, and behaviour change. The stems “communicat-”, “counsel-” and “modif-”, as well as the words “change” and “behaviour change”, were used to search all identified NLOs. 

## 3. Results

This section forms a descriptive analysis of the findings in this study. The testing of statistical significance was limited to Spearman’s Rank Correlation Coefficients (*Rs*).

### 3.1. Quantifying Nutrition-Related Learning Objectives

**Figure 1:**
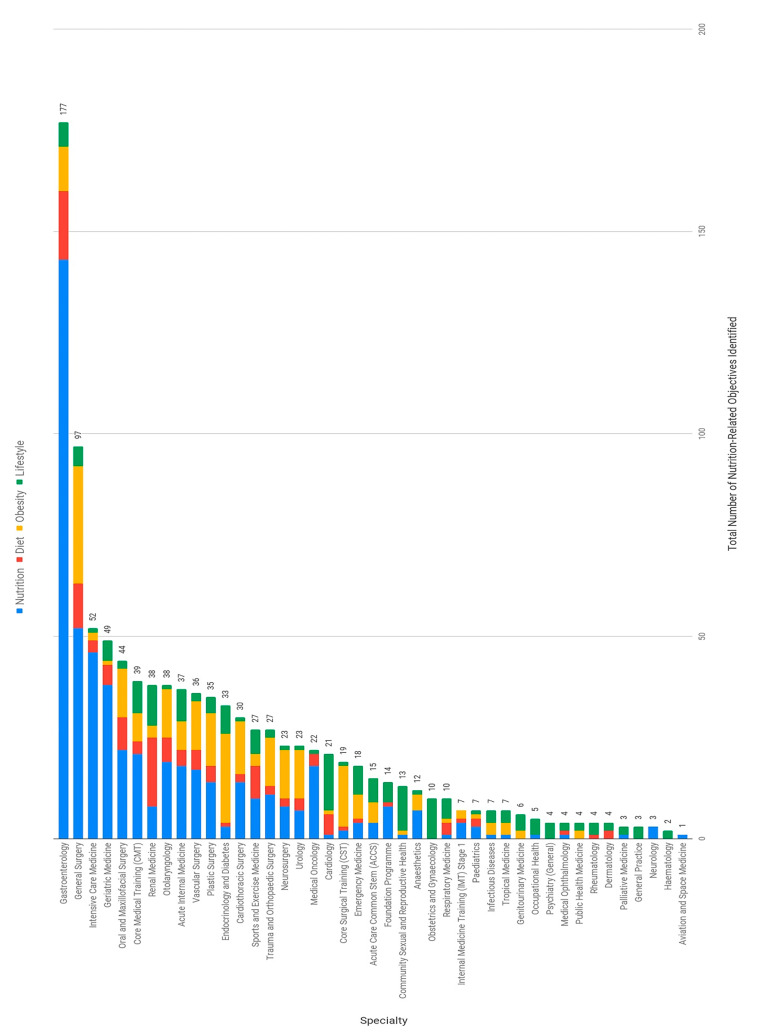
Total number of nutrition-related learning objectives identified per specialty (descending order), and the proportion of keywords used.

All 43 curriculums had at least one NLO identified, with a median of 15 NLOs (mean 24) per curriculum. There was considerable variation between curriculums in the number of NLOs identified (Figure 1), with a maximum of 177 (Gastroenterology) and a minimum of one (Aviation and Space medicine) (range = 176). Those curriculums with five or less NLOs ranked in the lower quartile (Q_1_); those in the upper quartile (Q_3_) had between 15 and 35 NLOs (interquartile range (IQR) = 30). The eleven curriculums in the lower quartile are listed in Table 2.

Surgical curriculums (*n* = 11) had a higher number of identified NLOs (median 30, mean 35 objectives) compared with medical curriculums (median 8.5 objectives, mean 20), and the number of NLOs identified in the IMT curriculum (7 objectives) was lower than the CMT curriculum (39 objectives). 

### 3.2. Comparison of Keywords Used

**Figure 2:**
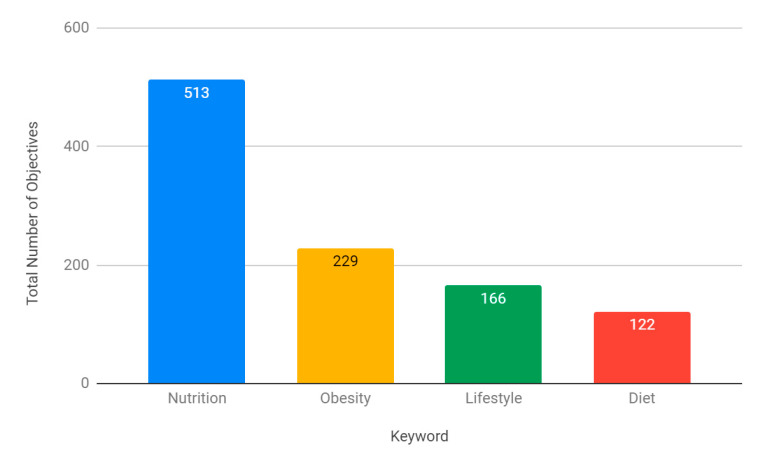
Total number of nutrition-related learning objectives identified per keyword across all curriculums.

Across specialties, the most common keyword in identified NLOs was ‘nutrition’ (513 objectives), followed by ‘obesity’ (229), ‘lifestyle’ (166), and ‘diet’ (122) (Figure 2).

### 3.3. Nutrition-Related Modules

In 22 specialties (51%) at least one nutrition-related module was identified, with a maximum of 13 modules in a single curriculum (Gastroenterology). Modules were identified in 100% of surgical curriculums (mean 2.7 modules per curriculum) compared with 34.4% of medical curriculums (mean 0.8 modules per curriculum). Shared peri-operative nutrition and obesity modules were present across all surgical curriculums, which likely contributes to the higher number of NLOs identified in surgical compared with medical curriculums. 

**Figure 3:**
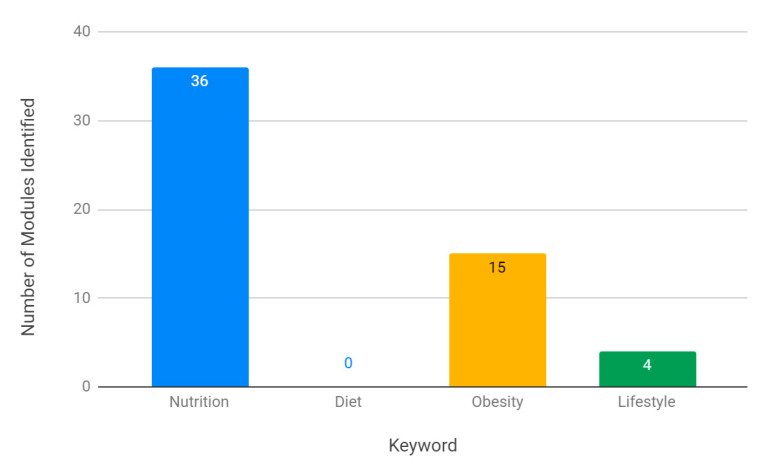
Number of nutrition-related modules per keyword across specialties.

The most common keyword descriptor for modules was ‘nutrition’ (36 modules across curriculums), followed by ‘obesity’ (15 modules) and ‘lifestyle’ (4 modules). There were no ‘diet’ modules identified in any curriculum (Figure 3).

### 3.4. Generic Professional Capabilities (GPC) Framework: Knowledge-, Skill-, or Behaviour-Based Outcomes

**Figure 4:**
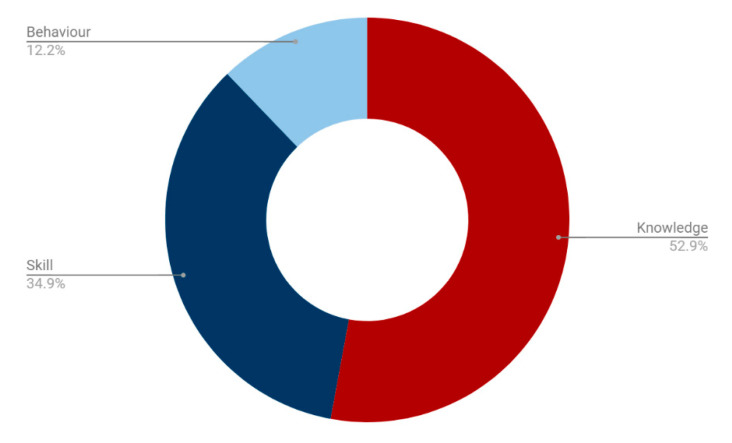
Proportion of nutrition-related objectives designated as knowledge-, skill-, and behaviour-based outcomes.

84% of NLOs identified across curriculums were designated as knowledge-, skill-, or behaviour-based. 52.9% of these were knowledge-based, compared with 34.9% skill-based and 12.2% behaviour-based objectives (Figure 4).

**Figure 5:**
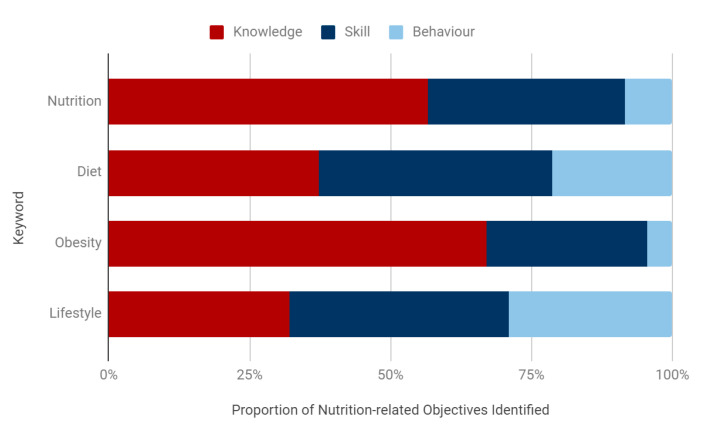
Proportion of knowledge-, skill-, and behaviour-based outcomes per keyword across specialties.

Over half of ‘nutrition’ and ‘obesity’ objectives were knowledge-based, with a small proportion of behaviour-based objectives (8.4% and 4.4%, respectively) (Figure 5). In comparison, ‘diet’ and ‘lifestyle’ objectives had the highest proportion of behaviour-based outcomes (21.3% and 29.1%, respectively) though these outcomes still remained a minority. ‘Diet’ objectives had the highest proportion of skill-based outcomes (41.5%). 

### 3.5. Good Medical Practice (GMP) Domains

Across curriculums, 51.9% of NLOs identified were designated one or more GMP Domains. Twelve specialties did not include any clear GMP Domain designation to NLOs, and were not included in this sub-analysis (General Surgery, Vascular Surgery, Plastic Surgery, Trauma and Orthopaedic Surgery, Neurosurgery, Community Sexual and Reproductive Health, Obstetrics and Gynaecology, Internal Medicine Training (IMT), Paediatrics, Occupational Health, Public Health, and General Practice). 

**Figure 6:**
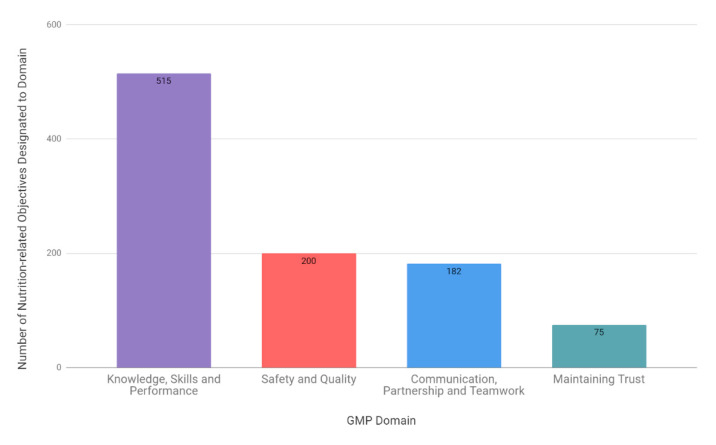
The number of nutrition-related objectives designated per Good Medical Practice (GMP) Domain across specialties.

The most commonly designated domain across curriculums was Domain 1: Knowledge, Skills and Performance (53.0%), followed by 2: Safety and Quality (20.6%), 3: Communication, Partnership and Teamwork (18.7%), and 4: Maintaining Trust (7.7%) (Figure 6). 

Spearman’s Rank Correlations were used to assess the relationship between keywords and GMP Domains (Table 3). These correlations indicated that there was a very weak positive association between ‘nutrition’ NLOs and Domain 1: Knowledge, Skills and Performance (*Rs*(31) = 0.19, *p* = 0.30), and very weak negative correlations with Domain 2: Safety and Quality (*Rs*(31) = −0.06, *p* = 0.74), Domain 3 (*Rs*(31) = −0.06, *p* = 0.75), and Domain 4 (*Rs*(31) = −0.08, *p* = 0.69). There was a weak negative correlation between ‘diet’ NLOs and Domain 1 (*Rs*(31) = −0.22, *p* = 0.23) and very weak positive correlations with Domain 2 (*Rs*(31) = 0.13, *p* = 0.49) and Domain 4 (*Rs*(31) = 0.23, *p* = 0.22). There was a moderately strong positive correlation between ‘diet’ NLOs and Domain 3: Communication, Partnership and Teamwork (*Rs*(31) = 0.41, *p* = 0.02). There was a weak negative correlation between ‘obesity’ NLOs and Domain 1 (*Rs*(31) = −0.22, *p* = 0.23) and Domain 2 (*Rs*(31) = 0.30, *p* = 0.1), a weak positive correlation with Domain 3 (*Rs*(31) = 0.29, *p* = 0.17) and a very weak negative correlation with Domain 4 (*Rs*(31) = −0.06, *p* = 0.73). ‘Lifestyle’ NLOs had a very weak positive correlation with Domain 1 (*Rs*(31) = 0.03, *p* = 0.87) and Domain 4 (*Rs*(31) = 0.07, *p* = 0.70), a weak negative correlation with Domain 2 (*Rs*(31) = −0.20, *p* = 0.29) and a very weak negative correlation with Domain 3 (*Rs*(31) = −0.01, *p* = 0.94). 

### 3.6. Communication Keyword Search

A search for the stem “communicat-” across all objectives revealed only eight NLOs, outlining communication-based outcomes with either members of the multidisciplinary team or patients (0.008% of 1030 identified NLOs). The stem “counsel-” appeared in 3 NLOs (0.003%), and “modif-” appeared in 13 NLOs (0.01%). “Change” appeared in 59 NLOs (0.06%) and was associated with lifestyle change in all but one objective; the full term “behaviour change” was found in one objective (0.001% of objectives). 

## 4. Discussion

### 4.1. Main Findings

This study provides a comprehensive critical synthesis of nutrition-related education in UK postgraduate medical training by identifying nutrition-related learning objectives (NLOs) and their designation within the GPC and GMP Domain frameworks. Using this standardised, quantitative approach we found that inclusion of NLOs is highly variable across curriculums, with a difference of 176 objectives between the highest and lowest scoring specialties (Figure 1). The results of this study support that the generic NLOs set out by the Nutrition Task Force in 1994 [26] have not been systematically incorporated into postgraduate curriculums, as 51% of specialties failed to incorporate a minimum of at least 18 objectives. In fact, a quarter of specialties had fewer than five (Table 2). 

Although the median number of objectives was found to be close to the Nutrition Task Force standard, at 15 NLOs per curriculum, the considerable variability across training programmes suggests that further work is required to ensure consistency in nutrition-related education across specialties. In particular, unsubstantiated differences between surgical and medical curriculums—with surgical specialties incorporating a median of 30 NLOs compared with 8.5 NLOs in medical curriculums—highlight the need for further evaluation of nutrition-related competencies across medical training programmes. 

Surgical programmes were also ahead of their medical counterparts in the inclusion of modules. All surgical curriculums contained at least one nutrition-related module (with an average of 2.7 per curriculum), whilst only 34.4% of medical curriculums managed the same (with an average of 0.8 modules per curriculum). Shared peri-operative nutrition and obesity modules were present across all surgical curriculums, and suggest that incorporation of shared, generic modules across medical curriculums may facilitate the inclusion of a greater number of NLOs, as well as promoting a cohesive approach to nutrition education. 

Using the GPC and GMP Domain frameworks to understand the specific contexts in which nutrition-related objectives appear within curriculums, it is clear that there is a predominance of knowledge-based outcomes. Within the GPC framework, 52.9% of NLOs were knowledge-based (Figure 4), which was highly consistent with the GMP Domain framework, where 53% of NLOs were designated as Domain 1: Knowledge, Skills and Performance (Figure 6). The communication skills and professional behaviours fundamental to effective patient care and successful behaviour change in nutritional care remain in the minority. This is particularly highlighted by the finding that only 20.6% of NLOs were assigned to Domain 2: Safety and Quality, 18.7% were assigned GMP Domain 3: Communication, Partnership and Teamwork, and 7.7% to Domain 4: Maintaining Trust (Figure 6). This is despite the fact that, unlike the GPC framework, more than one GMP Domain can be assigned to a single NLO, suggesting a true deficiency in these areas. Finally, a search for words relating to communication skills and behaviour modification strategies revealed that less than 0.1% of all identified NLOs directly addressed these competencies. 

Interestingly, an analysis of the keywords used within NLOs suggests that their wording may be associated with their designation within both the GPC and GMP Domain frameworks. In the GPC framework, over half of the ‘nutrition’ NLOs were knowledge-based, with a small proportion of behaviour-based objectives (8.4%) (Figure 5). ‘Diet’ objectives, on the other hand, had the highest proportion of skill-based outcomes (41.5%), and the second highest proportion of behaviour-based outcomes (21.3%). These findings were again mirrored within the GMP Domain frameworks, with ‘nutrition’ NLOs being weakly associated with Domain 1: Knowledge, Skills and Performance, and ‘diet’ objectives being moderately, and statistically significantly, associated with Domain 3: Communication, Partnership and Teamwork (Table 3). These findings are particularly important as no curriculums were found to incorporate a ‘diet’ module (Figure 3), signifying a specific area for improvement that might facilitate the inclusion of fundamental communication skills and professional behaviours. 

### 4.2. Nutrition-Related Objectives and Their Variation across Specialties

Learning objectives set out in postgraduate curriculums form the essential component upon which doctors will be taught, trained, and assessed throughout their training programme. Postgraduate programmes are required to ensure that doctors have sufficient experience, teaching, and training—including attending organised education sessions, training days, and courses—to meet the requirements of their GMC-approved curriculum [18]. Workplace-based assessments (WBAs) and postgraduate examinations must be mapped to the requirements of the curriculum [18]. Some postgraduate examinations may also derive their syllabus exclusively from the relevant specialty training curriculum; for example, Membership of the Royal Colleges of Physicians of the United Kingdom (MRCP(UK))—a mandatory examination for general medical trainees in the UK [30]—tests the acquisition of knowledge, skills, and behaviour as specified in the curriculum for Core Medical Training/Internal Medicine Training [31]. As such, the finding that the new IMT curriculum contained far fewer NLOs than the CMT curriculum it will replace (39 and 7, respectively), suggests that the representation of nutrition in this exam may be detrimentally affected. Finally, trainees must be barred from progressing through training if they fail to meet the required learning outcomes set out in their approved curriculum [18]. 

For trainees to be effectively taught, trained, and assessed on nutrition-related competencies, it seems clear that a sufficient number of nutrition-related learning objectives must be consistently incorporated into postgraduate curriculums. In light of the recognition that nutrition education is often waylaid by its sporadic and piecemeal inclusion throughout curriculums, objectives would ideally be integrated using a cohesive, modular approach, in order to facilitate learning and to champion the significance of nutrition in medical training. 

### 4.3. The Generic Professional Capabilities (GPC) Framework

In looking to increase the representation of nutrition-related learning objectives across postgraduate curriculums in a meaningful way, it is also essential that we examine the contexts in which these objectives appear. For UK postgraduate curriculum development, this largely refers to three fundamental areas—professional knowledge, professional skills, and professional values and behaviours—that form the foundation of the Generic Professional Capabilities (GPC) Framework set out by the GMC [19]. The GPC Framework has been embedded into postgraduate curriculums by assigning learning objectives as knowledge-, skill-, or behaviour-based outcomes. A full description of the framework is set out in the GPC document available on the GMC website [19], but in summary: knowledge-based outcomes relate to keeping up to date with medical knowledge and meeting the standards expected of all doctors, skill-based outcomes relate to practical skills such as clinical assessments, communication, and interpersonal skills, and behaviour-based outcomes require trainees to demonstrate appropriate personal and professional behaviours, such as acting with honesty and integrity, maintaining patient trust, acting without discrimination or prejudice, and being a professional role model [24]. 

All three of these GPC components are critical in nutrition-related training for doctors. Traditionally, however, acquisition of knowledge has formed the principal driving force of medical education. Despite the relationship between effective clinical communication and improved health outcomes being well established in the literature by the 1990s [32], communication competencies were not systematically incorporated into medical school curriculums in the UK until 2003 [33]. Since then, the significance of communication-based approaches in effectively managing patients, including in those with nutrition-related risk factors or disease, has been increasingly recognised [34]. The need for such skills is aptly summarised in the NHS statement that doctors should “feel equipped to talk to [patients] about… achieving a healthy weight in an informed and sensitive way” [3]. To achieve this goal, a strong foundation in clinical knowledge is required to understand concepts in practice, high-level communication skills are essential in providing effective patient care and facilitating successful behaviour change, and professional values, such as building empathetic, trusting, and respectful relationships with patients, are integral to a therapeutic relationship. Consequently, training curriculums must champion skill- and behaviour-based outcomes, as well as knowledge-based objectives, in relation to nutrition education. 

Although a lack of nutrition knowledge is an identified barrier to nutrition counselling [15], knowledge transfer alone is not sufficient to modify eating behaviours [35]. Dietician-patient interactions that heavily rely on information-giving have been shown to lead to high patient drop-out in comparison to motivational counselling approaches [36], and the majority of successful interventional weight loss trials have included behavioural modification as part of the intervention arm [37,38]. It is therefore essential that doctors are assessed on, and supported in achieving, skill-based competencies to engage with behavioural motivational counselling techniques. In this study, however, ‘obesity’ objectives across curriculums had the lowest proportion of behaviour- and skill-based outcomes, and the highest proportion of knowledge-based outcomes—a concerning finding given evidence that didactic, knowledge-based obesity interventions are less effective than motivational behaviour modification techniques [39].

### 4.4. Good Medical Practice (GMP) Domains

A second important GMC framework embedded into postgraduate curriculums is Good Medical Practice (GMP) [18]. There are four GMP Domains, which outline the standards expected of a doctor to justify the trust that patients place in healthcare professionals. Domain 1: Knowledge, Skills and Performance, describes the need to provide a good standard of practice and care by keeping professional knowledge and skills up to date, and recognising and working within the limits of your competence. Domain 2: Safety and Quality, describes the need to take prompt action if patient safety, dignity, or comfort is compromised, and also to protect and promote the health of patients and the public. Domain 3: Communication, Partnership and Teamwork, focuses on treating patients as individuals and respecting their dignity and right to confidentiality, working in partnership with patients by listening and responding to their concerns and preferences, providing information they want or need in a way they can understand, and supporting patients in caring for themselves to improve and maintain their health. Domain 3 also encompasses working well with colleagues, in ways that best serve patients’ interests. Domain 4: Maintaining Trust, is about acting with honesty and integrity, never discriminating unfairly against patients or colleagues, and never abusing the trusting relationship between the healthcare profession and the public. 

As with the GPC Framework, all of these domains are important in nutrition-related clinical practice. Whilst maintaining a good standard of practice and keeping up to date with knowledge and skills are fundamental, training curriculums should also focus on protecting and promoting the health of patients and the public (Domain 2), communicating effectively with patients and supporting them in improving and maintaining their health (Domain 3), and never discriminating unfairly against patients (Domain 4), within their nutrition-related training and education. 

Professional values, such as building empathetic, trusting and respectful relationships with patients are integral to successful behaviour change [35]. Weight stigmatization, prevalent in medical practice [40], is an impediment to effective weight management in patients. Personal values are also important, as doctors are more likely to counsel their patients if they practice healthy habits themselves, and patients are also more likely to listen [41]. Even clinicians attempting to improve poor habits counselled patients more often than those not trying to change their own behaviour [42]. Demonstrating personal nutritional values is a form of leadership and role-modelling, and doctors should be encouraged and supported to act as health champions in order to provide better care for patients. In order to make substantive changes in both clinical practice and medical education, we must emphasise and champion nutrition-related skills and behaviours [15].

### 4.5. A Matter of Patient Safety

The Nutrition Task Force and Intercollegiate Group on Nutrition recognised the need for nutrition-related education and training as a prerequisite to safe and competent clinical practice [22]. To illustrate why curriculums with a low number of NLOs—particularly relating to diet and obesity—are unlikely to ensure the competency of trainees in clinical practice, we provide examples from Core Psychiatry, Paediatrics, and General Practice. Whilst many medical specialties (e.g., haematology, neurology, dermatology) had low-scoring curriculums, trainees in those specialities benefit from training pathways that necessitate achievement of Core Medical or Acute Care Common Stem competencies. Core Psychiatry, Paediatrics, and General Practice—in addition to caring for vulnerable patient populations—require trainees only to complete the Foundation Programme prior to specialisation [43], further limiting their exposure to nutrition-related outcomes.

#### 4.5.1. Core Psychiatry

Individuals with depression and schizophrenia have a higher level of obesity than the general population, and are disproportionately at risk of obesity-related conditions including cardiovascular disease and type 2 diabetes mellitus (T2DM) [44,45]. Furthermore, antipsychotic medication side effects of weight gain and dyslipidaemia contribute to higher levels of obesity and T2DM [45]. Research indicates that inadequate attention to modifiable risk factors, such as a BMI > 25, increases the risk of cardiovascular disease in mental health patients [44]—the leading cause of death in this population [46]. 

The National Institute of Mental Health identified obesity as a priority [44,47]; however, the Core Psychiatry curriculum, which outlines the competencies that trainees must demonstrate in order to progress onto sub-specialty training [48], had only four identified NLOs. There were no nutrition, diet, or obesity-specific objectives identified. Objectives currently require trainees to “demonstrate an understanding of factors that influence the aetiology and course of mental disorder, and “advise on environmental and lifestyle changes”, “be aware of potential personal prejudices”, and be able to “advise patients about environmental and lifestyle factors and the adverse effects of alcohol, tobacco and illicit drugs”. These non-specific objectives do not ensure that trainees have the knowledge, skills or behaviours to facilitate discussions with patients about diet or obesity. Research by the Royal College of Psychiatrists states that in over 2000 research papers “we were disappointed to find very few examples of interventions used to control or prevent obesity”, with staff reporting a “lack of knowledge and confidence in the area of nutrition and obesity management” as a barrier to intervention [49]—suggesting that psychiatric training would benefit from specific nutrition-related competencies. 

#### 4.5.2. Paediatrics

In this study, we identified seven paediatric NLOs. ‘Nutrition’ objectives required trainees to “provide nutritional support for patients, including those with temporary or permanent intestinal failure, and manage services for home parenteral nutrition”, “manage all aspects of reversible and irreversible intestinal failure in children, and manage children with complex nutritional needs requiring nutritional support”, and “feeding and nutrition” in neonatal medicine. ‘Diet’, ‘obesity’ and ‘lifestyle’ objectives were to “work closely with…dieticians”, “liaise effectively with…specialist teams for managing paediatric inherited metabolic conditions, particularly...dieticians”, management of “endocrine-related obesity”, and playing “a vital role in planning and implementing local strategies to improve the health of all children in their area, including…targeted lifestyle programmes”. There are no competencies which specifically address diet and obesity counselling for children and parents—which have been shown to improve prevention and treatment of paediatric obesity [50]—nor any that are specific to dietary management of paediatric T2DM and its complications. 

The Royal College of Paediatrics and Child Health acknowledge that paediatricians have a role in the full breadth of child health. The management and prevention of childhood obesity is fundamental to a healthy lifestyle; overweight children are more likely to suffer psychosocial problems which affects educational attainment and interpersonal relationships and lead to persistent problems in adulthood [51,52]. In addition, a third of children leaving primary school are now classified as overweight or obese [2,3], and overweight/obese children are also more likely to become obese adults with consequent adverse health outcomes [53]. With the obesity epidemic, there has been an increase in T2DM among children and adolescents [54], with evidence suggesting that macro- and microvascular complications, such as stroke, myocardial infarction, sudden death, chronic renal failure, limb-threatening neuropathy and vasculopathy, and vision-threatening retinopathy, occur at younger ages in individuals who develop childhood T2DM [54]. This suggests that inclusion of diet and obesity competencies is required to support paediatric trainees and their patients. 

#### 4.5.3. General Practice

With the growing prevalence of non-communicable diseases (NCDs), General Practitioners (GPs) play an important role in the future trajectory of population health [43]. Over 60 million people in the UK are registered with a general practice [55], making GPs ideally placed to promote healthy nutrition and physical activity [56]. In the UK, 93% of those at risk of, or suffering from, malnutrition are living in the community [1], and improving the identification and treatment of malnutrition is estimated to have the third highest potential to deliver cost savings to the NHS [1]. 

The role of GPs in tackling the increasing burden of obesity and NCDs is recognised by the Royal College of GPs (RCGP) [43]. This study, however, found only three NLOs in the current, approved curriculum. There were no competencies relating specifically to nutrition, diet, or obesity, nor any nutrition-related modules identified. GP NLOs require trainees to “consider the impact of the patient’s lifestyle on his or her health”, “provide individually tailored, evidence-based advice and support to enable each patient to optimise his or her lifestyle and well-being”, and to “adopt counselling, motivational and behaviour change techniques when appropriate, prompting patients to reflect on the benefits of lifestyle change and support them to improve their health and self-care” [43]. These objectives are essential to individualised, evidence-based behaviour change management, but as generalists GPs are required to have understanding of nutrition-related objectives across a range of medical and surgical specialties [43], such as indications for enteral/parenteral feeding, or perioperative nutrition and weight optimisation. 

It is also crucial that GPs have skills and tools to manage obesity and diet with patients. The attendant COVID-19 (Severe Acute Respiratory Syndrome Coronavirus 2) crisis illustrates the importance of addressing malnutrition in primary care. A meta-analysis found substantial increases in morbidity and mortality associated with COVID-19 in patients with obesity, with 113% higher risk of hospitalisation, 74% higher risk for intensive care admission, and 48% increase in mortality [56]. The role of nutrition in the management of COVID-19 is heterogeneous, but underlying risk factors for severe COVID-19 infection, such as fatty liver disease, hypertension, dyslipidaemia, and T2DM, are preventable, primarily managed in the community, and all significantly associated with obesity and suboptimal diet [56]. New government campaigns to tackle obesity acknowledge COVID-19 as a “call to action” [57], and aim to facilitate and incentivise community-based interventions aimed at weight loss and healthy diet, empowering GPs to play a critical role in lifestyle modification [57]. However, without emphasis on nutrition-related competencies established in postgraduate medical training, government initiatives are unlikely to be meaningful or sustainable.

### 4.6. Strengths and Limitations

To our knowledge, this is the first study to quantitatively assess nutrition-related outcomes in postgraduate training in the UK. Using this study design, we were able to identify relevant NLOs and compare variation across specialties. We have built a database of all current nutrition-related outcomes that can be identified by ‘nutrition’, ‘diet’, ‘obesity’, and ‘lifestyle’ descriptors, which should provide the foundation for future qualitative work. In addition, we were able to assess the balance of knowledge-, skill-, and behaviour-based competencies, as well as Good Medical Practice Domains, to allow for continuing improvements toward GMC-aligned goals for excellence in postgraduate training programmes. 

Study limitations include the quantitative focus of this study design. Firstly, the number of NLOs in a curriculum bears no reflection on actual nutrition-related practice within an identified medical field, and we are therefore unable to comment on how variation in nutrition-related outcomes in curriculums translates into variation in practice. However, this data can serve as a starting point for further research seeking to understand the impact of curricular design on clinical practice. Secondly, a full thematic analysis of all objectives was outside the scope of this study. Future qualitative research into postgraduate training curriculums NLOs coupled with the findings in this study may help inform the development of new, high-level outcomes. Finally, in postgraduate training there is an assumption that all curricular competencies will be achieved, but recognised that not all may be specifically assessed, meaning that it is possible that, regardless of the number of NLOs in a curriculum, trainees may have evidenced competence in a smaller number of outcomes. In curriculums where NLOs constitute a very small proportion of total objectives, it may be possible for a trainee to progress through training without any demonstrated competence in this area. Given the concern regarding nutrition-related education and training for UK medical students and doctors, future research might look into the necessity for compulsory workplace-based assessments relating to nutrition-related outcomes. 

In addition, the exclusive inclusion of the current, GMC-approved curriculum for each specialty was necessary to ensure standardisation, but it should be recognised that some specialties, such as General Practice, also include detailed supplementary documents for trainees in which further reference to nutrition-related education may be present. However, the RCGP state “The core curriculum statement provides a full description of the knowledge, skills, attitudes and behaviours required of a GP in managing patients and their problems”, while the topic guides illustrate “key learning points with case scenarios, reflective questions, and advice for learning and teaching” [58], indicating that the curriculum alone should allow meaningful appraisal of the competencies expected of a trainee in a given specialty.

## 5. Conclusions

Patient health and safety is at the core of the educational standards in postgraduate medical training, and should accordingly be the first priority of organisations that design and develop curriculums [20]. Action-oriented research should be used to address identified barriers which hinder doctors in recognising and effectively treating malnutrition, a major cause of mortality and morbidity in the UK [1,2,3,4,5]. The findings in this study suggest the need for a standardised approach to nutrition education, which fundamentally integrates a generic set of nutrition-related objectives across all postgraduate medical curriculums, in line with GMC frameworks for excellence. This study provides evidence of the need for renewed focus on communication skills and professional behaviours to ensure that all doctors feel equipped to help patients achieve or maintain a healthy diet and weight, in an effective, informed and sensitive way. We must emphasise and champion nutrition-related education in postgraduate training, for our future doctors, and for our patients.

## Figures and Tables

**Table 1:** Description of Good Medical Practice (GMP) Domains [29].

GMP Domain	Description
1	Knowledge, Skills and Performance
2	Safety and Quality
3	Communication, Partnership and Teamwork
4	Maintaining Trust

**Table 2:** Specialty curriculums in the bottom quartile, with five or less nutrition-related learning objectives (NLOs) (descending order).

Specialty Curriculum	Number of NLOs Identified
Occupational Health	5
Core Psychiatry	4
Medical Ophthalmology	4
Public Health Medicine	4
Rheumatology	4
Dermatology	4
Palliative Medicine	3
General Practice	3
Neurology	3
Haematology	2
Aviation and Space Medicine	1

**Table 3:** Associations between keywords and Good Medical Practice (GMP) Domains across curriculums, demonstrated using Spearman’s Correlation Coefficient (*Rs*). * *p* = 0.1, ** *p* = 0.02.

		Keyword		
GMP Domain	Nutrition	Diet	Obesity	Lifestyle
1	0.19	−0.22	−0.22	0.03
2	−0.06	0.13	0.30 *	−0.20
3	−0.06	0.41 **	0.29	−0.01
4	−0.08	0.23	−0.06	0.07

## Appendix A

The The 18 fundamental learning objectives set out by the Nutrition Task Force [22], reproduced from Jackson, A.A., 2001. Human nutrition in medical practice: the training of doctors. Proceedings of the Nutrition Society, 60(2), pp. 257–263 [21].

### Appendix A.1. Principles of Nutritional Science

- Diets, foods and nutrients (substrates and cofactors)
- Metabolic demand, digestion and absorption, balance and turnover, physical activity, metabolic effects of excess, obesity
- Requirements, essentiality, bioavailability, limiting nutrients, effects of nutritional status on biochemical and organ function
- Adaptation to low nutrient intakes, body composition (form and function)
- Assessment of diet and nutritional status
- Physiological mechanisms that determine appetite, sociological, psychological, economic and behavioural aspects of food choice

### Appendix A.2. Public Health Nutrition

- The average British diet, including subgroup differences (e.g., region, gender, ethnic origin), lifestyle, risk factors and epidemiology (socio-economic factors, smoking and activity)
- Pre-conception, pregnancy, breast-feeding, infant nutrition, growth and development, ageing
- Dietary reference values, dietary recommendations and guidelines, diet and CHD and stroke, the health targets
- Nutritional surveillance and identification of markers of nutritional status
- Achieving change, education and motivation (education resources, theory and skills)
- Food supply, monitoring, cost–benefit of nutritional interventions, legislation, food labelling and policy which affects food consumption

### Appendix A.3. Clinical Nutrition and Nutritional Support

- Assessment of clinical and functional metabolic state, effect of functional state on nutritional intake and status, effect of status on clinical outcomes
- Anorexia and starvation, response to injury, infection and stress
- Altered nutritional requirements in relevant disease states, unusual requirements
- General principles of nutritional support, routes of support
- Basis of nutrition-related diseases, therapeutic diets (diabetic, renal), weight reduction
- Drug–nutrient interactions

## Appendix B

List of specialty curriculums which were not included in this study:

- Allergy
- Audio vestibular medicine
- Chemical pathology
- Child and adolescent psychiatry
- Clinical genetics
- Clinical neurophysiology
- Clinical oncology (medical oncology used)
- Clinical pharmacology and therapeutics
- Clinical radiology
- Diagnostic neuropathology
- Forensic histopathology
- Forensic psychiatry
- Histopathology
- Immunology
- Medical microbiology
- Medical psychotherapy
- Medical virology
- Nuclear medicine
- Old age psychiatry
- Ophthalmology (curriculum not available in a standard format on the GMC website, medical ophthalmology curriculum used in lieu)
- Paediatric and perinatal pathology
- Paediatric cardiology
- Paediatric surgery
- Pharmaceutical medicine
- Psychiatry of learning disability
- Rehabilitation medicine

## Appendix C

Potential keywords identified using Medical Subjects Headings (MeSH) tree structures in the National Institutes of Health (NIH) database:

- Obesity
- Body weight
- Weight gain
- Weight loss
- Overweight
- Nutrition
- Enteral/parenteral nutrition
- Bariatrics
- Bariatric surgery
- Gastric Bypass
- Gastroplasty
- Jejunoileal Bypass
- Lipectomy
- Appetite
- Satiation/satiety
- Food
- Meals
- Hunger
- Feed/feeding
- Nutritive value
- Glycaemic index
- Glycaemic load
- Diet
- Cooking
- Energy intake
- Caloric restriction
- Fasting
- Portion size
- Serving size
- Eating
- BMI (body mass index)
- Body fat distribution
- Adiposity
- Body composition
- Body size
- Waist circumference
- Lipid accumulation product
- Sagittal abdominal diameter
- Waist-height ratio
- Body surface area
- Skinfold thickness
- Waist-hip ratio
- Biometry
- Anthropometry
- Kinanthropometry
- Calories
- Caloric restriction
- Malnutrition
- Overnutrition
- Fat
